# Association between lithium in tap water and suicide mortality rates in Miyazaki Prefecture

**DOI:** 10.1186/s12199-020-00865-6

**Published:** 2020-06-27

**Authors:** Naomi Kozaka, Shouhei Takeuchi, Nobuyoshi Ishii, Takeshi Terao, Yoshiki Kuroda

**Affiliations:** 1 Department of Public Health, Faculty of Medicine, University of Miyazaki, 5200 Kihara, Kiyotake, Miyazaki, 889-1692 Japan; 2 Department of Nutrition Science, Faculty of Nursing and Nutrition, University of Nagasaki, 1-1-1 Manabino, Nagayo, Nishisonogi, Nagasaki, 851-2195 Japan; 3 Department of Neuropsychiatry, Oita University Faculty of Medicine, 1-1 Idaigaoka, Hasama-machi, Yufu, Oita 879-5593 Japan

**Keywords:** Elderly people, Japan, Lithium, Rainfall, Suicide rate, Tap water

## Abstract

**Background:**

Most studies have reported that suicide mortality rates are negatively associated with lithium levels in tap water; however, a few studies showed either no association or a positive association. Thus, the association between suicide mortality and lithium levels in tap water remains controversial. To clarify the association, our study evaluated the association between lithium levels in tap water and suicide mortality rates in Miyazaki Prefecture of Japan, after adjusting for confounding factors.

**Methods:**

We measured lithium levels in tap water across the 26 municipalities of Miyazaki Prefecture in Japan. We examined the standardized mortality ratio (SMR) for suicide in each municipality and used the data as the average suicide SMRs over 5 years (2009–2013). Weighted least-squares regression analysis, adjusted for the size of each municipality’s population, was used to investigate the association between lithium levels in tap water and suicide SMRs. In addition to a crude model, in an adjusted model, potential confounding factors (proportion of elderly people, proportion of one-person households, annual marriage rate, annual mean income, unemployment rate, the density of medical doctors per 100,000 people, annual total rainfall, and proportion of people with a college education or higher) were added as covariates.

**Results:**

We showed that male and female suicide SMRs were not associated with lithium levels in tap water in Miyazaki Prefecture. After adjusting for confounders, male suicide SMRs were significantly and positively associated with the proportion of elderly people in the population and annual total rainfall, and female suicide SMRs were associated with the proportion of elderly people in the population.

**Conclusions:**

No association between lithium levels in tap water and suicide mortality rates was found in Miyazaki Prefecture.

## Background

Suicide is a serious problem in Japan, and the World Health Organization has reported that Japan has the highest suicide mortality rates of any country [1]. Furthermore, Japan’s suicide mortality rates increased suddenly following the economic crisis at the end of the twentieth century. The number of suicide victims in Japan was over 30,000 in 1998, and the number remained over 30,000 every year afterwards, until 2011. Recently, suicide mortality rates in Japan have been declining; however, the number of suicide victims in 2016 was still around 20,000. Although suicide mortality rates decreased from 26.0 per 100,000 population in 1998 to 17.3 per 100,000 population in 2016 [2], this rate is still higher than developed countries in Europe and the USA.

Suicide is a complex public health problem involving mental health issues, and the causes of suicide are complicated. Age, gender, amount of sunshine, economic situation, and psychological states, as well as cultural, social, biological, genetic, and environmental factors could all be associated with suicide [3–5]. A previous study indicated that within a national cohort of Denmark, the risk of suicide was higher among individuals with psychiatric disorders than those without [6].

Regarding the effects of these factors on the suicide mortality rates in Japan, it can vary widely among the country’s 47 prefectures. Generally, the suicide mortality rates in Japan tend to be higher in northern areas of the country. In the Kyushu area, located in the southern part of Japan, the suicide mortality rates of each prefecture are relatively lower than in other areas, except for in Miyazaki Prefecture. The suicide mortality rates in Miyazaki Prefecture were 23.4 per 100,000 population in 2015, which was higher than the mean of that year for the whole of Japan of 18.5 per 100,000 population [7]. The detailed causes of this high suicide rates are not well understood.

In an ecological study performed in 1990, Schrauzer and Shrestha reported that suicide mortality rates were negatively associated with lithium levels in drinking water in 27 counties in Texas [8]. Ohgami et al. also reported a negative association between lithium levels in drinking water and suicide mortality rates for 18 municipalities in Oita prefecture in Japan [9]. After these findings were published, studies were performed in Austria, Aomori prefecture of Japan, 34 prefectures in Greece, and nine cities in Lithuania, and each showed a negative association between lithium levels in drinking water and suicide mortality rates [10–13]. Moreover, Pompili et al. showed a partial negative association between lithium levels in drinking water and suicide mortality rates among females in 145 sites in Italy [14]. Recently, Ishii et al. extended their investigation from one prefecture to Kyushu Island (a total of eight prefectures) and found that lithium levels in drinking water were negatively associated with male suicide mortality rates, after adjustment for potential confounding factors [15]. However, two studies, one in England and one in Portugal, indicated no association between lithium levels in drinking water and suicide mortality rates [16, 17]. Knudsen et al. performed a study using individual-level, register-based data for the entire adult Danish population (3.7 million individuals) from 1991 to 2012, linked with a moving 5-year time-weighted average (TWA) with adjusted spatial autocorrelation (i.e., neighboring observations tend to be more alike compared to those further apart). As a result, they found that the suicide mortality rates increased along with an increasing 5-year TWA lithium exposure level [18]. Therefore, the relationship between suicide mortality rates and lithium levels in tap water remains controversial.

Lithium has been effectively used in the treatment of bipolar disorder, and increasing evidence suggests its effectiveness in reducing the risk of suicide. Several meta-analyses have shown anti-suicidal effects in people with mood disorders, such as clinical depression and bipolar disorder [19–21]. However, lithium doses used for mental health treatment are considerably higher than those obtained from daily exposure to lithium in tap water, which raises questions regarding whether daily intake of lithium from tap water can reduce the risk of suicide. Therefore, we investigated the association between lithium levels in tap water and suicide mortality rates in Miyazaki Prefecture after adjusting for confounding factors. We verified whether lithium levels in tap water can lower the suicide rates in Miyazaki Prefecture, as the suicide mortality rates in Miyazaki Prefecture has been the highest in Japan.

## Methods

### Study population

Miyazaki Prefecture is located in the Kyushu district of Japan. Population sizes vary widely across the prefecture’s 26 municipalities, with the largest population in the city of Miyazaki (402,572) and the smallest in the village of Nishimera (1199).

### Suicide mortality rates

We used the standardized mortality ratio (SMR) for each municipality, with regard to the differences in gender and age distributions in individual municipality populations. The SMR is an indirect method of adjusting mortality rates, defined as the number of observed deaths in an individual municipality’s population divided by the number of expected deaths, compared with gender and age matched general population. We obtained suicide mortality rates from the Ministry of Health, Labor and Welfare and used the average male and female suicide SMRs for 5 years (2009–2013), across all 26 municipalities in the prefecture.

### Lithium levels in tap water

We obtained tap water samples from water treatment plants located in Miyazaki Prefecture’s 26 municipalities. In 2013, the samples were collected in clean 200-ml plastic containers provided by a laboratory, which were then transported back to the laboratory. In total, 78 samples (ranging from 1 to 16 per municipality) of tap water from the public water supply systems of 26 municipalities were obtained and analyzed.

The lithium levels in the tap water samples from each municipality were measured using inductively coupled plasma-mass spectrometry at a certified laboratory (Saishunkan Reassurance and Safety Laboratory Co., Ltd. Kumamoto, Japan), after acidifying the water (HNO_3_:0.01% concentration) without sample filtration after collection. This method is used to measure very low levels of lithium, with a detectable minimum level of 0.1 ppb (0.1 μg/L). In this study, if lithium levels in tap water were measured from multiple water sources within the same municipality, the mean value was calculated. The distribution of lithium levels was considerably skewed (skewness, 2.96; kurtosis, 10.41). Thus, we employed log transformation (skewness, 0.12; kurtosis, − 0.52) to use parametric statistical procedures.

### Adjustment factors

We considered area-based sociodemographic characteristics that could be confounding factors associated with suicide, such as proportion of elderly people [4, 22, 23], proportion of one-person households [24], annual marriage rate [23–25], annual mean income [24, 26], unemployment rate [24, 26], density of medical doctors per 100,000 people [10], annual total rainfall [22, 23, 26–28], and proportion of people with a college education or higher [25]. The data regarding the proportion of elderly people in 2010, proportion of one-person households in 2010, annual marriage rate in 2013, annual mean income in 2013, unemployment rate in 2010, density of medical doctors per 100,000 people in 2010, and proportion of people with a college education or higher in 2010 were available for all 26 municipalities from the Statistics Bureau, Ministry of Internal Affairs and Communications. Since the data concerning the annual total rainfall in 2012 were not available from the Japan Meteorological Agency for all 26 municipalities, we used assumed rainfall data based on nearby municipalities for missing rainfall data.

### Statistical analysis

Due to significant differences in population sizes across the prefecture’s 26 municipalities, weighted least-squares regression analysis was used to adjust the size of each population to investigate the association between lithium levels in tap water and suicide SMR. A crude model was conducted to evaluate the relationship between lithium levels in tap water and suicide SMR. Regarding the adjusted model, multiple linear regression analysis was conducted to estimate the association between lithium levels and suicide SMR, adjusted for confounding factors. Multiple regression analyses were performed using stepwise procedures, which first treated lithium levels as an independent variable, and then included all confounding factors as independent variables. A value of *p* < 0.05 was considered significant. Data analysis was performed using SPSS 18.0.0 for Windows.

## Results

The mean suicide SMRs were 123 (SD, 50; range, 42–257) for the total population, 127 (SD, 47; range, 58–234) for males, and 114 (SD, 74; range, 0–312) for females in Miyazaki Prefecture. The mean lithium levels in the tap water were 2.8 μg/L (SD, 3.1; median, 1.7 μg/L; range, 0.2–12.3), and the mean log-transformed lithium levels in the tap water were 0.19 (SD, 0.48; range, − 0.7 to 1.09). The mean rates for confounding factors were as follows: proportion of elderly people was 30.9% (SD, 6.0), proportion of one-person households was 25.7% (SD, 3.7), annual marriage rate was 4.2% (SD, 0.9), annual income per person was ¥2,363,385 (SD, ¥151,392), unemployment rate was 6.2% (SD, 2.2), density of medical doctors per 100,000 people was 142 (SD, 69), proportion of people with a college education or higher was 7.2% (SD, 2.4), and annual total rainfall was 3,583 mm (SD, 443 mm).

**Fig. 1:**
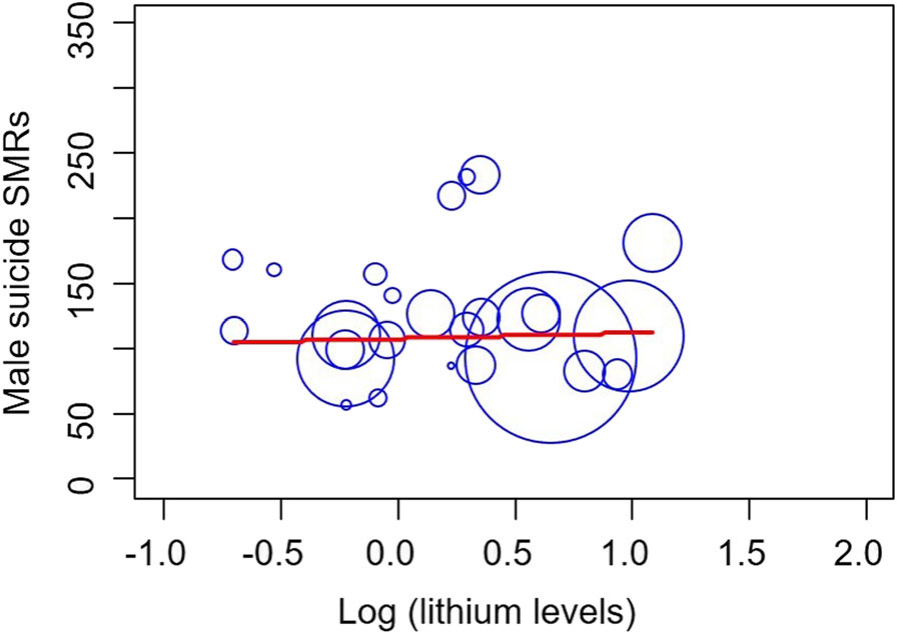
Lithium levels in drinking water and male suicide SMRs. The lithium levels are log-transformed, and the size of the dot represents population size. Male suicide SMRs were not associated with lithium levels (*β* = 0.061, *p* = 0.769)

**Fig. 2:**
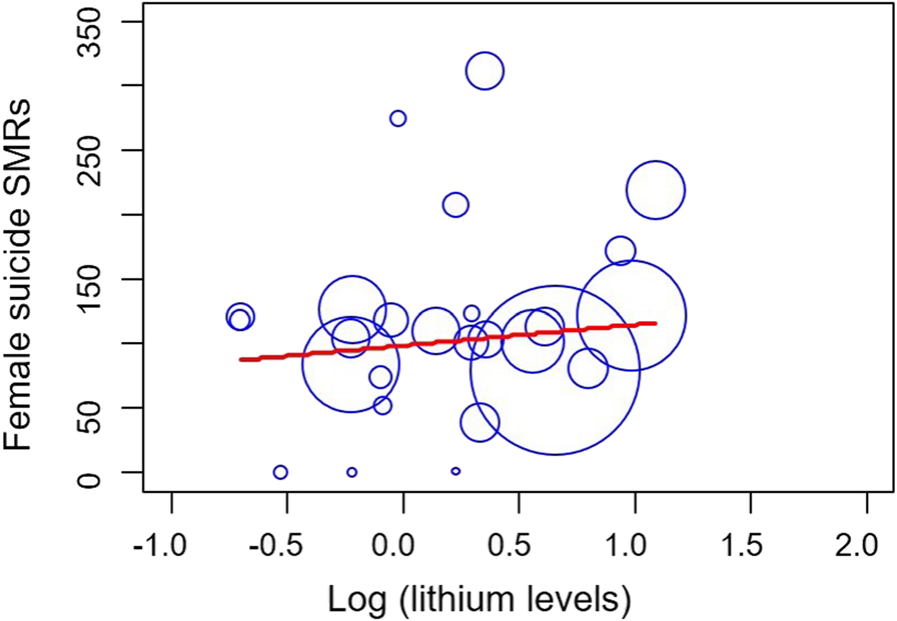
Lithium levels in drinking water and female suicide SMRs. The lithium levels are log-transformed, and the size of the dot represents population size. Female suicide SMRs were not associated with lithium levels (*β* = 0.154, *p* = 0.452)

Male and female suicide SMRs were not associated with lithium levels (males, *β* = 0.061, *p* = 0.769; females, *β* = 0.154, *p* = 0.452) in the crude model (Figs. 1 and 2).

Furthermore, we evaluated the associations between SMRs and the lithium levels as adjusted by confounders (Tables 1 and 2). In the adjusted model, male and female suicide SMRs were also not associated with lithium levels in tap water. However, we detected significant and positive associations between proportion of elderly people and annual total rainfall and suicide SMRs among males, as well as proportion of elderly people and suicide SMRs among females.

**Table 1:** Multiple regression analysis of male suicide SMRs and lithium levels in drinking water

Male	*β*	*p*	Adjusted *R*^2^
Crude model			
Log lithium levels	0.061	0.769	− 0.038
Adjusted model			
Log lithium levels	0.303	0.06	0.475
Proportion of elderly people	0.545	0.002	
Proportion of one-person households	0.005	0.979	
Annual marriage rate	− 0.035	0.249	
Annual mean income	− 0.158	0.534	
Unemployment rate	− 0.072	0.672	
Density of medical doctors per 100,000 people	− 0.064	0.774	
Annual total rainfall	0.374	0.029	
Proportion of people with a college education or higher	− 0.041	0.885

**Table 2:** Multiple regression analysis of female suicide SMRs and lithium levels in drinking water

Female	*β*	*p*	Adjusted *R*^2^
Crude model			
Log lithium levels	0.154	0.452	− 0.017
Adjusted model			
Log lithium levels	0.313	0.075	0.34
Proportion of elderly people	0.628	0.001	
Proportion of one-person households	− 0.099	0.655	
Annual marriage rate	− 0.249	0.477	
Annual mean income	− 0.289	0.308	
Unemployment rate	0.004	0.984	
Density of medical doctors per 100,000 people	− 0.178	0.476	
Annual total rainfall	0.295	0.101	
Proportion of people with a college education or higher	− 0.34	0.28	

## Discussion

We investigated the associations between lithium levels in tap water, possible confounding factors, and suicide mortality rates across the 26 municipalities of Miyazaki Prefecture. Overall, our findings indicated no association between lithium levels in tap water and suicide mortality rates. This finding was not consistent with the findings of previous studies performed across 18 municipalities of Oita prefecture [9] and across 274 municipalities of Kyushu Island (eight prefectures, including Miyazaki Prefecture) [15].

In this study, the mean and range of lithium levels in the tap water samples (mean, 2.8 μg/L; range, 0.2–12.3 μg/L) were lower than those found in other studies (range, 0.7–59 μg/) [9], (mean, 4.2 μg/L; range, 0–130 μg/L) [15]. Further, compared to previous studies that indicated no association between lithium levels in tap water and suicide mortality rates, a study in England [16] found a lower range (< 1–21 μg/L) similar to our study; however, another study conducted in Portugal found higher mean and range of lithium levels (mean, 10.88 μg/L; range, < 1–191 μg/L) [17]. In that study, the researchers considered that they found no correlation between lithium levels and suicide mortality rates because the suicide mortality rates of Portugal were lower than those in other studies, the unemployment rate was high, and the mean income was low. They posited that because economic difficulties can increase suicide rates, the effect of lithium in tap water may be relatively small. Therefore, we consider that we could not detect a relationship between lithium levels in tap water and suicide SMRs because of the low concentration of lithium in the tap water samples. In addition, the lithium in tap water may be ineffective in lowering suicide rates because the contribution of lithium to suicide is insignificant comparing other factors such as psychosocial, economic, and meteorological ones, and the effect of lithium could not be detected.

It is not clear why the concentration of lithium in Miyazaki Prefecture is low. Dawson et al. reported a negative association between lithium levels in drinking water and annual rainfall in Texas [29]. Our study also showed a negative correlation between lithium levels in drinking water and annual total rainfall (*r* = − 0.529, *p* = < 0.001; data not shown). Lithium is transferred from rocks and soil into drinking water by rainfall; therefore, lithium levels in drinking water may be negatively related to rainfall diluting drinking water supplies [30]. Annual total rainfall in Miyazaki Prefecture was high, which could be a reason why the mean lithium levels of tap water were lower than those of previous studies.

**Fig. 3:**
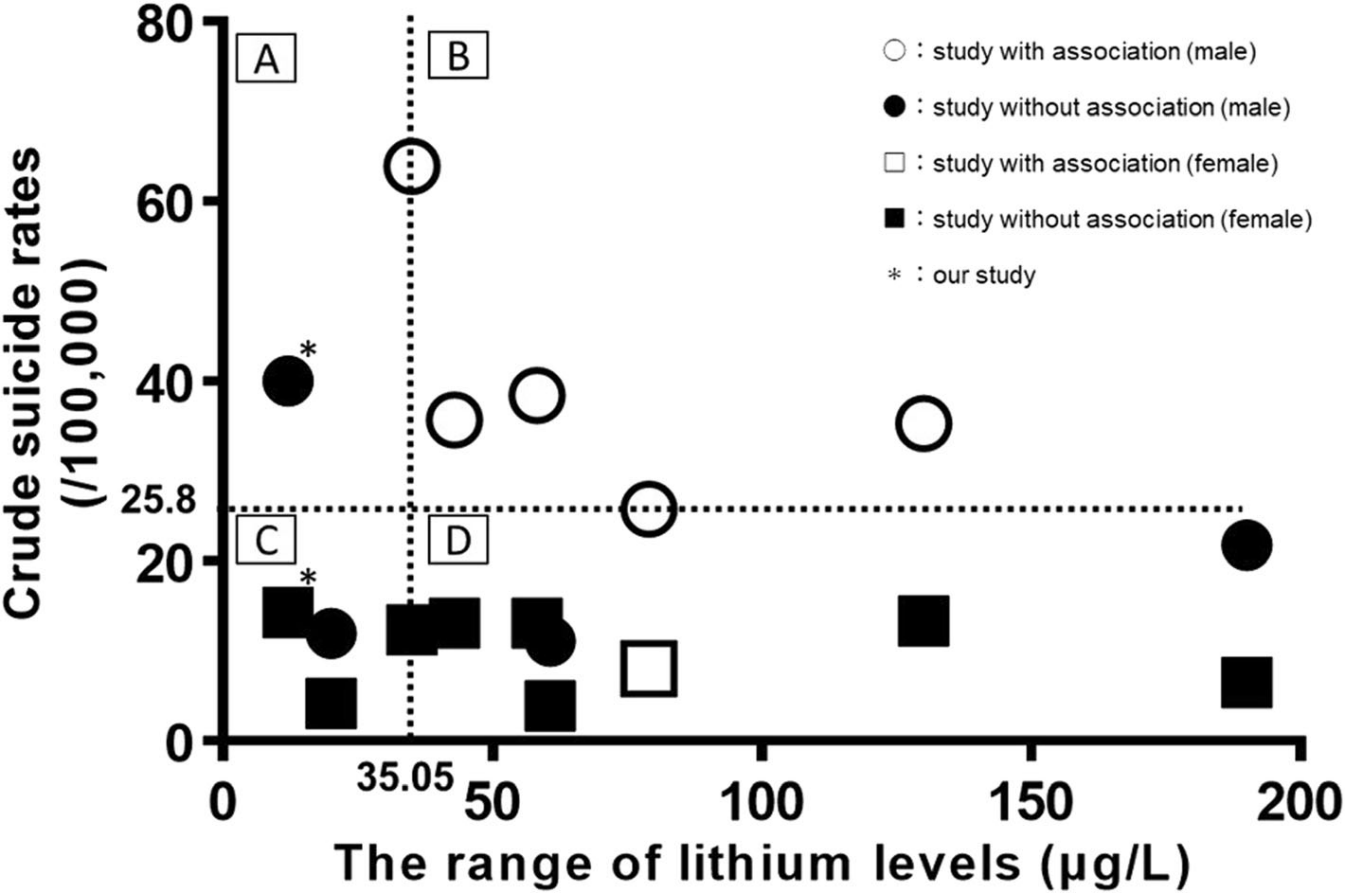
The range of lithium levels in drinking water and crude suicide rates (per 100,000 population). Studies with an association: these studies showed a significant association between lithium levels in tap water and suicide; if lithium levels increased, suicide rates decreased. Studies without an association: these studies did not show a significant association between lithium levels in tap water and suicide. The range of lithium levels: max value minus min value. The dotted lines show minimum levels of the range of lithium levels and crude suicide rates among studies with an association (male). Areas A, B, C, and D are divided by dotted lines (the horizontal line shows crude suicide rates 25.8/100,000, and the vertical line shows the range of lithium levels 35.05 μg /L). We used lithium levels from previous studies [9, 10, 13–17, 31], as well as crude suicide rates (per 100,000 population) from statistics on suicide provided by the Ministry of Health, Labor and Welfare in Japan and WHO [32]. If crude suicide rates of the survey year were not available, we substituted crude suicide rates of the nearest survey year

We speculated that the relationship between lithium levels and suicide mortality rates could affect differences in our study compared to the findings of previous studies regarding this association. Therefore, we evaluated the relationship between the range of lithium levels and crude suicide rates, separating them according to studies with an association (research that showed a significant association between lithium levels in tap water and suicide; if lithium levels increased, suicide rates decreased) and studies without such an association (research that did not show a significant association between lithium levels in tap water and suicide). We used lithium levels that were used in previous studies [9, 10, 13–17, 31] as well as crude suicide rates (per 100,000 population) from statistics on suicide provided by the Ministry of Health, Labor and Welfare in Japan and WHO [32]. Figure 3 shows the range of lithium levels in drinking water and crude suicide rates from eight previous studies and that of our study. With reference to previous research [10, 13], we divided Fig. 3 into four areas; the dividing lines for crude suicide rates and the range of lithium levels were 25.8/100,000 and 35.05 μg/L, respectively. Studies in Area B (crude suicide rates were over 25.8/100,000, and the range of lithium levels were above than 35.05 μg/L) reported that higher lithium levels were associated with lower suicide rates. However, almost none of the articles in Areas A, C, and D indicated any such association. Thus, as shown in Fig. 3, at relatively high suicide rates (crude suicide rates were over 25.8/100,000) and the higher range of lithium levels (lithium levels were above than 35.05 μg/L), an association between lithium in tap water and suicide rates could be detected. Conversely, if suicide rates were low (crude suicide rates were under 25.8/100,000) or the range of lithium levels were low (lithium levels were less than 35.05 μg/L), there may not be an association between lithium in tap water and suicide rates (as was also found in our study). However, the lithium concentration required in drinking water to lower suicide rates is unknown, and the association between lithium in drinking water and suicide rates as a dose-response relationship is also unclear. Thus, further studies are necessary to clarify this relationship.

We also evaluated other factors possibly associated with suicide rates. Our study showed a significant positive association between rainfall levels and suicide rates among males, which is in line with the findings of previous studies [23, 31]. Tsai further reported a significant positive association between rainfall levels and suicide rates among females in Taiwan [23]. Shiotsuki et al. reported a significant positive association between rainfall levels and suicide rates in males and the total population [31]. However, other studies reported significantly negative associations between suicide rates and rainfall levels [22, 26–28]. Thus, the relationship between suicide rates and rainfall levels may be controversial.

We detected a significant positive association between the proportion of elderly people in the population and suicide SMRs, which was in agreement with the findings of previous studies [4, 22, 23]. Suicide mortality rates increase with age, and the suicide rates of elderly people are high in Japan. It was reported that the majority of suicides were due to health problems, such as psychological issues and physical illnesses. Generally, elderly people are more likely to have health problems than younger people; thus, aging may be one of the risk factors for suicide.

Our study had some limitations. First, our findings were derived from local prefectures and, therefore, only limited generalization is possible. Second, lithium can be found not only in drinking water but also in food. Our study did not count dietary sources of lithium; therefore, it lacked data related to lithium levels in food and consumption habits of bottled mineral water. Third, other factors, such as prevalence and management of mental health disorders and migration of populations, were not taken into consideration. This was an ecological study that adjusted for limited sociodemographic variables; therefore, we could not adjust for unknown confounding factors. Moreover, concerning statistical methods, we used weighted least-squares regression analysis and multiple regression analysis, adjusting for confounding factors. However, spatial regression analysis, as performed by Knudsen et al. [18], could have been a more suitable method of analysis to investigate the effects of lithium exposure from tap water on suicide rates. Finally, the ecological nature of this study could not determine a causal relationship between lithium levels in tap water and suicide mortality rates. Thus, our results should be interpreted cautiously.

## Conclusions

No association between lithium levels in tap water and suicide mortality rates was found in Miyazaki Prefecture. However, we identified relationships among rainfall levels, the proportion of elderly people in the population, and suicide rates. Many possible factors are related to suicide mortality rates. Therefore, further studies are required to confirm the associations between lithium levels in drinking water, as well as other factors, and suicide mortality rates.

## Data Availability

The datasets used and analyzed concerning lithium levels in the current study are available from the corresponding author on reasonable request. The datasets generated and analyzed concerning study population, suicide mortality rates, and adjustment factors are publicly available from the Ministry of Health, Labor and Welfare, the Statistics Bureau, the Ministry of Internal Affairs and Communications, and the Japan Meteorological Agency.

## Abbreviations

SMR: Standardized mortality ratio
TWA: Time-weighted average
